# Update on drug development targeting parasite cysteine proteases

**DOI:** 10.1371/journal.pntd.0005850

**Published:** 2018-08-23

**Authors:** James H. McKerrow

**Affiliations:** Center for Discovery and Innovation in Parasitic Diseases, Skaggs School of Pharmacy and Pharmaceutical Sciences, University of California San Diego, La Jolla, California, United States of America; Washington University School of Medicine, UNITED STATES

As noted in the introductory review, cysteine proteases are attractive targets for structure-based drug design because of their well-defined active site topology and mechanism of action, available high-throughput assays, and known drugability.

Drugs targeting human cysteine proteases, homologous to those found in parasitic organisms, entered clinical trials as drug candidates for the treatment of psoriasis, osteoporosis, autoimmune diseases, and cancer. Specific cysteine proteases targeted by the pharmaceutical industry for drug development include cathepsin S, cathepsin K, and cathepsin B. Some of these development efforts are ongoing, others (e.g., Odanacatib) were halted for efficacy or toxicology reasons. Conversely, a number of chemical scaffolds and “warheads” have been evaluated as inhibitors of parasite cysteine protease targets [1]. While effective at the biochemical and cellular level, most of these were subsequently abandoned for efficacy or safety issues. Vinyl sulfones and nitriles, the focus of this update, remain viable after preclinical screens.

One can envision 2 approaches for targeting diseases caused by parasites. The first is the direct repurposing of drugs, developed and approved for treatment of host diseases against parasitic infections. Efforts to repurpose drugs developed against diseases thought to involve human cathepsin S are following this strategy. Safety is paramount, but failure of efficacy against a host disease is not always relevant.

The second approach focuses on specific active site-directed “warheads,” already proven efficacious for targeting other disease processes involving homologous cysteine proteases. An example of this approach is the exploration of nitrile derivatives (Fig 1) developed for osteoporosis as drugs to treat Chagas disease [2]. Nitrile-derivatized inhibitor scaffolds, while inhibiting cysteine proteases in a mechanism-based manner, are considered “reversible,” although often with slow off rates.

**Fig 1:**
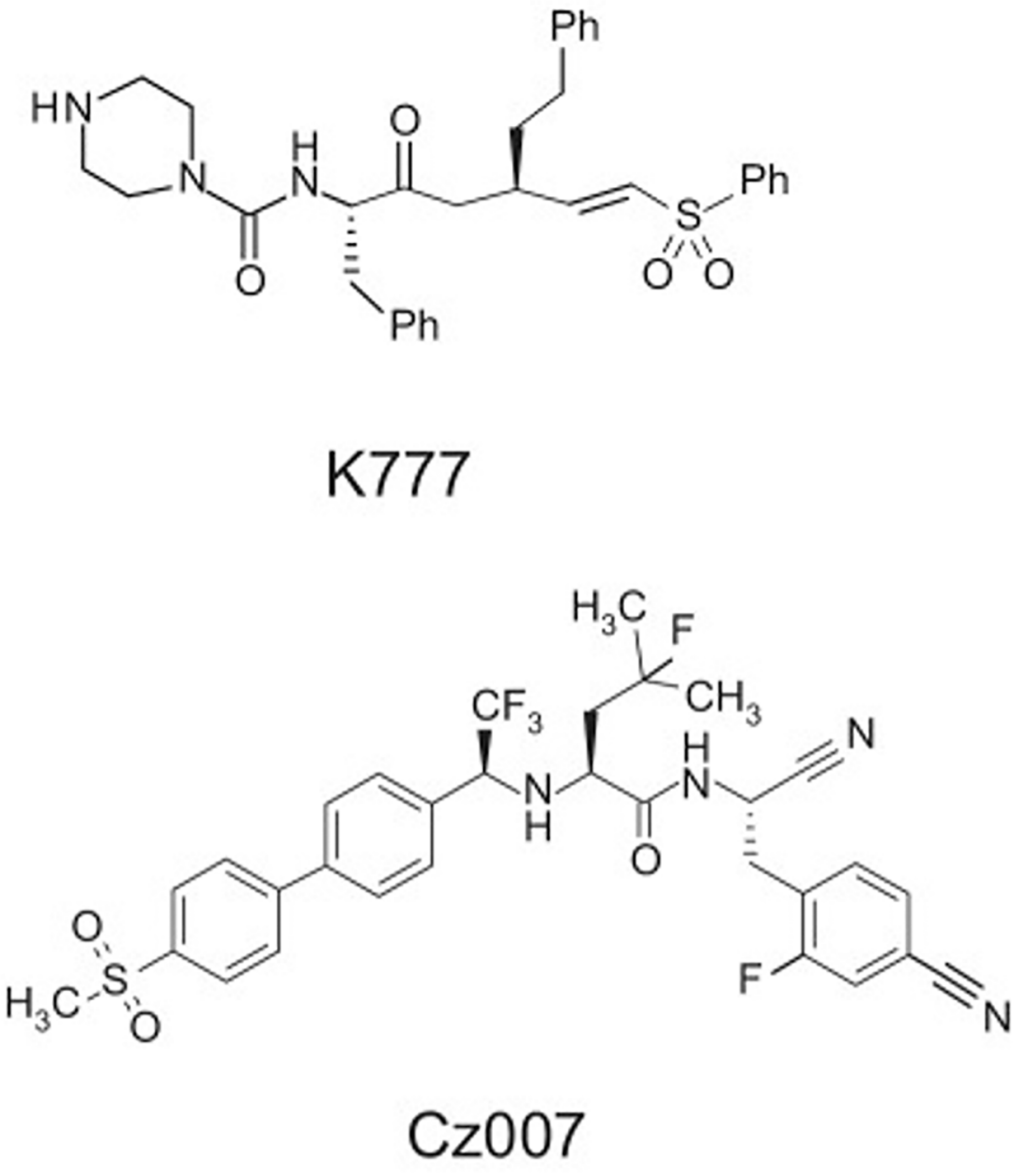
Structure of vinyl sulfone K777 and nitrile Cz007 [2].

Indeed, compounds that were abandoned early in the drug development process for failure to ameliorate a host disease may still have efficacy versus parasitic infections. An example is the “proof of concept” vinyl sulfone inhibitor K777 (also known as K 11777). This compound (Fig 1) was originally developed as an inhibitor of human cathepsin S by scientists at Khepri Pharmaceuticals [3]. K777 was effective in animal models of Chagas disease, schistosomiasis, hookworm infection, and cryptosporidiosis (referenced elsewhere in this PLOS NTDs collection). It may very well have other uses yet to be defined. Based upon its efficacy against the cysteine protease (cruzain, also known as cruzipain) of *Trypanosoma cruzi*, this inhibitor underwent extensive preclinical testing at several Contract Research Organizations (CROs). Key studies are summarized in Table 1. Importantly, the cysteine protease inhibitor was found to be safe in rodents, dogs, and nonhuman primates. Safety testing included 7-, 14-, and 28-day chronic dosing. In addition, specific metabolites of this compound were identified in mammals.

**Table 1:** K777 preclinical studies.

**Proposed therapeutic dose**	50 mg/Kg Bid in rodents for 20 days, 1–5 micromolar Cmax.
**7-day non-GLP toxicity study in dogs (SRI study M138-00)**	K777 HCl was administered orally as a solid in gelatin capsules to 2 dogs (1 male and 1 female) at 100 mg/kg daily for 7 days. No mortality or morbidity was observed in this study.
**7-Day non-GLP Toxicity Study in Rats (SRI study M587-08)**	K777 HCl was well tolerated in male and female rats at doses of 50 or 150 mg/Kg/day when administered for 7 days. The target organ of toxicity appears to be the liver, but at the doses employed in this study, the effects were mild and transient and are not expected to be dose-limiting.
**Acute intravenous non-GLP toxicity study in rats (SRI study M001-98)**	K777 HCl administered IV to male and female Sprague-Dawley rats produced mortality and adverse clinical signs at 150 mg/Kg and higher.
**Biotransformation and in vitro metabolism (UCSF/Benet Lab, SRI study M602-08)**	Metabolic profiling was performed using liver microsomes from 5 species (Sprague-Dawley rat, beagle dog, New Zealand white rabbit, Cynomolgus macaque, and human). In all species, the 3 major metabolites were observed. In human liver microsomes, the N-oxide had the highest rate of formation, followed by the N-desmethyl metabolite and low levels of the hydroxyl metabolite.
**K777 toxicity in hepatocytes (SRI study B150-04)**	Rat hepatocytes more sensitive than monkey or human. Cytotoxic at 100 micromolar and higher at 48 hours.
**K777 toxicity in immortalized human hepatocytes (Bristol-Myers Squibb)**	IC50 for cytotoxicity >50 micromolar at 20 hours.
**Neuropharmacological profile in rats (Covance Report 256/001-D6146)**	No effects at 100 and 300 mg/Kg, minor changes at 1,000 mg/Kg.
**In vivo micronucleus assay (SRI study M602-08)**	Negative at 150 mg/Kg for 14 days per oram (po) in rats.
**L5178Y mouse lymphoma cell tk+/-6tk-/-gene mutation assay (MLA) (SRI study G058-99)**	No mutations at 300 micrograms/mL.
**GLP bacterial mutagenicity assay (SRI study G056-99)**	No mutations at doses up to 5,000 micrograms/plate.
**14-day GLP toxicity study in Sprague-Dawley rats (SRI study M602-08)**	No mortality or treatment-related adverse clinical signs were observed following the administration of K777 HCl at any dose level.
	There were no treatment-related adverse effects on hematology, urinalysis, or ophthalmology parameters following treatment with K777 HCl. There were various changes in hematology parameters and organ weights that were not considered attributable to K777 HCl administration. Changes in clinical chemistry parameters included increases in total protein, potassium, phosphorus, and creatinine and decreases in blood urea nitrogen. These changes were not considered biologically significant. No treatment-related histopathological abnormalities were noted.
**Pilot multiple dose safety/pharmacokinetic 7-day non-GLP study in cynomolgus monkeys (SRI study M158-01)**	No mortality or morbidity was observed following the administration of K777 HCl at 200 mg/Kg once daily for 7 days.
**Pharmacokinetics in NHPs at 200 mg/Kg**	K777 HCl showed a Cmax of 4.5 μg/mL at 4 hours (Tmax) and an AUC of 32.5 μg-h/mL. The half-life was calculated to be approximately 4 hours (Fig 1).
	Structure of vinyl sulfone, K777 [3], and nitrile [2].

A final note about this compound is a caveat for future preclinical studies. The original compound was an HCl salt. Therefore, a drug suspension in water or saline alone gave a pH as low as 3–4. This resulted in emesis in monkeys at 500 mg/Kg and occasional bouts of emesis at doses as low as 50 mg/Kg (Harlan Study S43281). As a result, one international agency halted development just prior to funding a 28-day chronic dosing study. However, when the pH of the solution was neutralized at a second CRO (SRI International), emesis ceased to be a problem and the 28-day study at 2 doses was carried out with funding from the European Union (FP7).

Another key observation was that this covalent inhibitor, although not biochemically selective, was safe in mammals. In other words, although the inhibitor was equally effective against mammalian cathepsin B, L, and S, it proved to be safe in rodents, dogs, and monkeys. This “biological selectivity” was likely due to a number of factors and is probably applicable to other mechanism-based cysteine protease inhibitors.

First, mammals have a redundancy of cysteine proteases, making it unlikely that inhibition of cathepsin B or cathepsin L alone, for example, would lead to toxicity. Furthermore, gene deletion studies have shown that loss of individual cysteine protease genes did not result in neonatal or infant mortality or other significant safety issues. Finally, it is estimated that the concentration of cathepsins in human cellular organelles, like lysosomes, may reach millimolar. It would therefore be impossible to effectively inhibit host cathepsin activity with an ingested drug.

## Conclusion

A final important note is that a drug treatment targeting parasite cysteine proteases would resemble an antibiotic dosing regimen rather than the lifelong “management” of a disease like diabetes. If the drug is to be given for 1, 5, 10, or even 20 days, it is unlikely to require the same safety profile as a drug one takes over their lifetime. Mechanism-based inhibitors like the nitriles [2] or covalent inhibitors like vinyl sulfones or epoxides can therefore be envisioned as potential drugs to treat parasitic infections. Recent reviews emphasized the importance of resurrecting programs developing covalent drug candidates because of their historical success and safety record [4,5].

## References

[1] McKerrow J Development of cysteine protease inhibitors as chemotherapy for parasitic diseases: insights into safety, target validation, and mechanism of action(1999) International Journal of Parasitology 29, 833–838. 10480720 10.1016/s0020-7519(99)00044-2

[2] Ndao M, Beaulieu C, Black WC, Isabel E, Vasquez-Camargo F, Nath-Chowdhury M, Massé F, Mellon C, Methot N, Nicoll-Griffith DA.(2013) Reversible cysteine protease inhibitors show promise for a Chagas disease cure. Antimicrob Agents Chemother. 2014;58(2):1167–78. 10.1128/AAC.01855-13 24323474 PMC3910870

[3] Palmer JT, Rasnick D, Klaus JL, and Bromme D(1995) Vinyl sulfones as mechanism-based cysteine protease inhibitors J.Med. Chem 38, 3193–3196. 10.1021/jm00017a002 7650671

[4] Singh J., et al. (2011) The resurgence of covalent drugs. Nature Reviews Drug Discovery 10, 307–317. 10.1038/nrd3410 21455239

[5] Skilos M, BenAissa M, Thatcher GRJ Cysteine proteases as therapeutic targets: does selectivity matter?(2015) Acta Sinica B 5, 506–519 10.1016/j.apsb.2015.08.001 PMC4675809 26713267

